# Key influences in the design and implementation of mental health information systems in Ghana and South Africa

**DOI:** 10.1017/gmh.2016.3

**Published:** 2016-04-08

**Authors:** S. Ahuja, T. Mirzoev, C. Lund, A. Ofori-Atta, S. Skeen, A. Kufuor

**Affiliations:** 1 Public Health Foundation of India, New Delhi, India; 2 Nuffield Centre for International Health and Development, University of Leeds, UK; 3 Department of Psychiatry and Mental Health, Alan J Flisher Centre for Public Mental Health, University of Cape Town, South Africa; 4 University of Ghana Medical School, Ghana

**Keywords:** Design, Ghana, health management information system, implementation, mental health, mental health information system, South Africa

## Abstract

**Introduction:**

Strengthening of mental health information systems (MHIS) is essential to monitor and evaluate mental health services in low and middle-income countries. While research exists assessing wider health management information systems, there is limited published evidence exploring the design and implementation of MHIS in these settings. This paper aims to identify and assess the key factors affecting the design and implementation of MHIS, as perceived by the key stakeholders in Ghana and South Africa.

**Methods:**

We report findings from the Mental Health and Poverty Project, a 5-year research programme implemented within four African countries. The MHIS strengthening in South Africa and Ghana included two related components: intervention and research. The intervention component aimed to strengthen MHIS in the two countries, and the research component aimed to document interventions in each country, including the key influences. Data were collected using semi structured interviews with key stakeholders and reviews of key documents and secondary data from the improved MHIS. We analyzed the qualitative data using a framework approach.

**Results:**

Key components of the MHIS intervention involved the introduction of a redesigned patient registration form, entry into computers for analysis every 2 months by clinical managerial staff, and utilization of data in hospital management meetings in three psychiatric hospitals in Ghana; and the introduction of a new set of mental health indicators and related forms and tally sheets at primary care clinics and district hospitals in five districts in the KwaZulu-Natal and Northern Cape provinces in South Africa. Overall, the key stakeholders perceived the MHIS strengthening as an effective intervention in both countries with an enhanced set of indicators in South Africa and introduction of a computerized system in Ghana.

**Discussion:**

Influences on the design and implementation of MHIS interventions in Ghana and South Africa relate to resources, working approaches (including degree of consultations during the design stage and communication during implementation stage) and the low priority of mental health. Although the influencing factors represent similar categories, more influences were identified on MHIS implementation, compared with the design stage. Different influences appear to be related within, and across, the MHIS design and implementation and may reinforce or negate each other thus leading to the multiplier or minimization effects. The wider context, similar to other studies, is important in ensuring the success of such interventions.

**Conclusion:**

Future MHIS strengthening interventions can consider three policy implications which emerged from our analysis and experience: enhancing consultations during the intervention design, better consideration of implementation challenges during design, and better recognition of relations between different influences.

## Introduction

Mental health is an important though still neglected area of public health around the world (Doku *et al*. 2008; Lund *et al*. 2008; Omar *et al*. 2010; WHO, 2010). Globally, the proportion of the global burden of disease attributable to mental illness increased by 37% between 1990 and 2010, to account for 7.4% of disability adjusted life years (Murray *et al*. 2012). The total cost of mental illness is the highest among non-communicable diseases and it is expected to increase by 41% from 2010 to 2030 (Bloom *et al*. 2012).

National health management information systems (HMIS) are well-recognized sources of information to inform decisions in relation to the health of populations (AbouZahr & Boerma, 2005). HMIS often includes programme specific information systems, such as mental health information systems (MHIS), which may be integrated into the general HMIS. As such, the objective of MHIS is to provide timely and accurate information to ensure the most appropriate approach to addressing mental health problems through service delivery. Over the last decade, MHIS strengthening (i.e. developing and improving MHISs which are integrated with the broader health information system of countries) has been emphasized globally as a means of improving the monitoring of mental health care (Lund & Flisher, 2003; WHO, 2005; Garrib *et al*. 2008). Different approaches to evaluate health information systems (HIS) exist. Examples of these include (a) framework and standards for Country Information Systems proposed by the Health Metrics Network of the WHO, which distinguished six components (HIS resources, indicators, data sources, data management, information products, dissemination and use) structured in three categories (inputs, processes and outputs) (HMN, 2008) and (b) good evaluation practice guidelines for Health Informatics, proposed by International Medical Informatics Association in collaboration with the European Federation for Medical Informatics, which differentiates six related Phases in the process (preliminary outline, study design, operationalization of methods, project planning, execution and completion of the evaluation study), (HNM, 2008; Nykänen *et al*. 2011; Brender *et al*. 2013). While such frameworks provide an excellent start, most studies progressing from these frameworks focus on assessment of wider HMIS (Gladwin *et al*. 2003; Chaulagai *et al*. 2005; Odhiambo-Otieno, 2005; Smith *et al*. 2008; Krishnan *et al*. 2010) and not programme-specific information systems such as MHIS. Despite the existence of methodological guidance on the design of MHIS (WHO, 2005), we found no studies exploring the views of key stakeholders in relation to the main factors influencing the design and implementation of MHIS in low and middle-income countries (LMICs). We aim to contribute to filling this gap through reporting our experiences of strengthening MHIS intervention in two African countries, Ghana and South Africa.

The main objective of this paper is to identify the key influences on the MHIS design and implementation in the two countries. While we outline the systems architecture to complement descriptions available elsewhere (Ofori-Atta *et al*. 2010, 2012) and briefly identify the main effects of the MHIS strengthening, we focus mostly on reporting key influences on the design and implementation of MHIS.

These study findings emanate from the mental health and poverty project (MHaPP), a 5-year (2005–2010) research programme consortium, which was implemented in Ghana, South Africa, Uganda and Zambia and involved nine institutional partners from Africa and Europe. The project aimed to raise the profile of the field and break the cycle of poverty and mental ill health through the partnerships between research teams and policy-makers from the ministries of health in the participating countries (Flisher *et al*. 2007; Omar *et al*. 2010). The MHaPP project was implemented in two phases: situational analysis, and implementation and evaluation of interventions. In the implementation phase, the strengthening of MHIS in Ghana and South Africa was identified by the research team following discussions with relevant ministries of health as one of the three priority areas or mental health strengthening (Lund *et al*. 2009; Ofori-Atta *et al*. 2010).

## Methods

### Study components

The MHIS strengthening in both countries included two related components: intervention and research. The intervention component aimed to strengthen the MHIS in each country, whereas the research component aimed to document and assess the implementation of interventions in each country, including the key influences.

A generic protocol – covering both intervention and research components – was developed and was subsequently adapted and used by each country team to guide the study in each country. This generic protocol outlined the key issues for consideration in MHIS design in each country, including the aim and objectives for the intervention and research components. This involved mapping the existing information, mechanisms and practices; consulting with stakeholders on the shape of the intervention; developing detailed system specifications including data collection forms; training of staff on implementation; implementing and monitoring.

The generic protocol also included detailed guidance on the research component, including advice on the data collection and data analysis methods as well as sample tools for consideration by the country teams.

#### Intervention component

In Ghana, the focus of the MHIS strengthening intervention was on all the country's three psychiatric hospitals: Accra, Pantang and Ankaful, whereas in South Africa, strengthening of district health information system (DHIS) was done in selected districts in two provinces: Northern Cape and KwaZulu-Natal. In both countries the MHIS strengthening was a joint initiative involving researchers from the MHaPP project and the relevant department within national or regional (provincial) Ministries of Health to ensure ownership and to design a system responsive to needs of its main users. As shown in Table 1, the *intervention component* included three sequential steps in each country: designing, implementation and monitoring and evaluation.

**Table 1.** Intervention steps in each country

Step	South Africa	Ghana
Design	• Developing a pilot set of indicators for integration into district health information system • Designing data collection forms and manuals • Resource planning for the intervention • Designing training and workshops	• Setting up of indicators for mental illnesses (following ICD-10) • Developing new patient registration forms, manuals and software for data entry • Developing training and workshops manuals • Resource planning for the intervention
Implementation	• Distribution of new forms and manuals • Training and workshops: ◦ Introduction, data collection and analysis; ◦ Feedback on data collected and its use in decisions; ◦ Refresher and troubleshooting • Continuous monitoring	• Distribution of new forms and manuals • Training and workshops: ◦ Introduction to and use of ICD 10 in diagnosis; ◦ Use of new forms; ◦ Data entry into the new software; ◦ Use of information in patient care, hospital management and advocacy decisions • Bi-monthly reporting and discussion of results at hospital meetings
Monitoring and evaluation	• Feedback meetings with key stakeholders • Evaluation of the effects	• Feedback meetings with key stakeholders • Evaluation of the effects

In South Africa, a task team was established, involving researchers and province-level policymakers, which agreed on the key principles for collaboration and identified the specific areas for MHIS strengthening in the two provinces (MHaPP, 2010 and MHaPP 2010*a*). The following MHIS strengthening principles, proposed by WHO, guided the design of the intervention: broad consultations, user-friendliness in the system, addressing the information requirements of the stakeholders, integrating the information systems and viewing it as a part of the wider health system (WHO, 2005). In South Africa, before MHaPP, one mental health indicator was captured in KwaZulu-Natal (mental health visit) and four in Northern Cape Province (mental health visit, new mental health visit, mental health visit by patient under 18 years of age, and number of patients on mental health register). The need for increasing the mental health indicators was identified by the task team. The new indicators were arrived at through a situation analysis and an extensive process of consultation with stakeholders, including the mental health programme staff, primary care staff and monitoring and evaluation staff in the Department of Health at provincial and district level. Once the new indicators were agreed on, the task team developed new manual data collection tools (forms and tally sheets). This was followed by training of staff in their use and subsequent workshops to ensure quality checks as part of the monitoring and evaluation.

In Ghana, the aim of the intervention was to strengthen the functions of collection, processing, analysis, dissemination and use of information in the three psychiatric hospitals. Discussions between the research team, doctors, administrators, records officers and Policy, Planning, Monitoring and Evaluation division within the Ghana Health Service identified the need for a computerized MHIS in all the psychiatric hospitals in Ghana (Ofori-Atta *et al*. 2010). The design of MHIS in these three psychiatric hospitals was also based on WHO guidelines (WHO, 2005). The design of the indicators, data collection forms for recording diagnoses of mental illnesses and software was guided by the International Classification of Diseases (ICD-10). To improve the quality (reliability, timeliness, comprehensiveness, accuracy) and breadth of data collected, a new computer software system was designed for data entry, with manuals defining variables to be collected. A new patient registration form was designed and piloted in all the three hospitals and changed to suit the information needs of the hospitals as staff at different levels of care and management was consulted repeatedly. Staffs were trained on data collection and entry, analysis and use of data in reports and planning. New records staffs were hired by two of the hospitals and National service personnel were deployed to increase capacity in the hospitals for data entry and analysis. New computers were purchased by MHaPP project for the three hospitals to facilitate data entry. Prescribers (Psychiatrists, Doctors and Medical Assistants) participated in several meetings on the data being collected and the role they played in filling out the patient forms, as well as what the preliminary data meant with respect to patient care. In order to improve the utilization of information for mental health planning, policy, monitoring and evaluation, managers were trained to use the data once it was collected, entered and analyzed. Similar to South Africa, training of hospitals’ health staff on the use of new forms was conducted and subsequent workshops were held to ensure quality checks and encourage the use of information in decision-making. The new system was allowed to run for a full year while being monitored regularly.

#### Research component

This study aims to document and assess the implementation of interventions in each country, including identification of main influencing factors. The respondents’ views and perceptions were used in identifying the key influencing factors on the MHIS design and implementation. This component is described in more detail in subsequent sub-sections.

### Study design

The main study design involved qualitative research on the process and key influences of implementing the new systems, involving in-depth interviews with key stakeholders as well as reviews of key documents related to MHIS in each country.

### Study sample

All study respondents were purposefully selected, using non-randomized purposive sampling, given the limited number of stakeholders who were involved in the design and implementation of the MHIS intervention in each country. Table 2 provides a detailed breakdown of number of respondents in each country. In Ghana, a total of 41 semi structured interviews were conducted with medical and paramedical staff, managers and records staff. In South Africa the total number of interviews held was 40 with 26 respondents, and included 14 repeat interviews for the different roles played by the same officials.

**Table 2.** Participants in semi-structured interviews in Ghana and South Africa

Respondent group	Role in MHIS	Number in each country	
		Ghana	South Africa
Province level managers/policy makers	Implementing national policies and developing province-level policies		4
Provincial level information systems	Managing health information system		5
Local level managers (district or facilities)	Managing service delivery	1	4
Medical records administrators (district or facilities)	Administering records	2	4
Mental health care personnel	Clinical services, collection of data at primary health care level	1	9
Hospital staff	Clinical services, collection of data at hospital level	37	
TOTAL		41	26

### Data collection

The data for this study were collected using two methods: in-depth interviews with key stakeholders and reviews of key documents. The in-depth interviews were the main data collection method in this study, and the findings from document reviews were used to triangulate the results and then analyse the data. Each of the two data collection methods is described next.

The generic protocol, referred to earlier, was adapted by the country teams in preparing for the data collection. The question guides for in-depth interviews were structured around: (a) issues related to processes of designing and implementing the intervention and (b) issues related to the effects of the system. The key influences on the design and implementation of the MHIS cut across these two categories of questions.

Individual in-depth semi-structured interviews were conducted with key respondents, approximately 1 year after the commencement of the implementation of the MHIS intervention. All interviews were audio-recorded, transcribed verbatim and used for analysis. Informed consent was obtained from all respondents prior to each interview.

The review of purposefully-selected documents covered key policies outlining and regulating the design of HMIS and MHIS in each of the two countries, as well as the MHaPP project reports. Minutes from task team meetings and teleconferences were also reviewed and results compared with findings from the interviews.

### Data analysis

A Framework Approach was used in each country to guide the analysis of results, which included stages of familiarization with the data, indexing and charting, coding of the interviews with the help of NVIVO software (which allow assigning the different codes to specific passages of interview transcripts), and mapping and interpretation of results (Miles & Huberman, 1994; Ritchie & Spencer, 1994).

Data were analyzed using inductive approach (i.e. allowing for themes to mainly emerge from the data) and in a step-wise manner by building a logical chain of evidence with themes emerging from the data after noting the patterns, metaphors and clustering in the text. The initial framework for indexing the data reflected the structure of adapted interview guides (separate sections or categories for: design and implementation phases, and for processes and effects of interventions). This framework was continuously updated using document reviews as the specific themes emerged from our analysis. The key influences which were emphasized by majority of respondents, and were mostly evident in the documents, were regarded as most important as compared with those raised by only some respondents and which rarely featured in the document. The themes were compared across the different groups of stakeholders and between the interviews and documents, to understand differences and similarities and ensure validity of our results.

To ensure validity of results, analysis of data was conducted by at least two researchers. Furthermore, results of analysis of data from the interviews were triangulated between the different respondent groups and with results of document reviews.

As part of analysis, factors which affected design and implementation of MHIS intervention in each country were identified and grouped into broad categories. Simplified versions of causal loop diagrams were used to illustrate and visualize the relationships between various factors. Causal relationships were identified by the researchers from the analysis of interview transcripts and documents.

Ethics approvals were obtained from the Human Research Ethics Committee, in the Faculty of Health Sciences, University of Cape Town (REC Ref: 314/2005) and the Ghana Health Service Ethical Review committee. All the data were stored securely and as mentioned earlier the interview transcripts were anonymized for analysis.

## Results

We now briefly outline the overall architecture of MHIS in each country and outline the main perceived effects, as a background for reporting the key perceived influences on the MHIS intervention in the two countries.

In each hospital in Ghana, the data collection on mental health was done manually, using a newly redesigned patient registration form (which included demographic details, patient's diagnoses, prescription and availability of prescription). Once completed, the data were then entered into computers for analysis. Statistics from the new system were analyzed every 2 months by clinical managerial staff, and fed into specially set up hospital management meetings. Although with a view of future integration, in parallel, the old system of registration of manual data collection producing monthly reports also continued.

In South Africa, the following individual indicators were added to the existing mental health indicators at each level:

• Primary health care level (including clinics and community health centres):
  ◦ Total mental health visits
  ◦ New mental health visits
  ◦ Number of mental health clients on register
  ◦ Number of mental health visits per broad diagnostic group: substance abuse, intellectual disability, anxiety disorder, mood disorder, psychosis
  ◦ Number of mental health visits for patients under 18 years of age
• Secondary care level (district hospitals):
  ◦ Outpatient indicators as per primary care level (above)
  ◦ Number of mental health admissions
  ◦ Number of mental health discharges
  ◦ Average length of stay (days)
  ◦ Number of admissions per diagnostic group: parasuicide, substance abuse, anxiety disorder, mood disorder, psychosis
  ◦ Adverse events
  ◦ Readmissions within 3 months of discharge.

At the primary care and district hospital levels, data were captured on pen and paper using tally sheets. These were then aggregated and captured electronically on a monthly basis by the District Information Officer. The District Information Officer then generated regular reports from the data in the DHIS system.

### Main perceived effects of MHIS intervention

Overall, the MHIS strengthening was perceived to be effective by the key stakeholders, particularly health staff and managers, in both countries (MHapp, 2010).

In Ghana, the uniform system of recording diagnoses of mental illnesses using the ICD-10 was piloted, which contributed to strengthened capacity of the records department (Ofori-Atta *et al*. 2010). Feedback from the staff after the first year of implementation revealed improved staff motivation. This was perceived to be crucial for mental health system development and helped the staff and managers to understand the importance of information for decision-making.

In South Africa, stakeholders interviewed expressed the opinion that availability of additional mental health indicators for programme planning and management has improved capacity of district management teams to collect quality information and ensure its use in decision-making.

…*I think with the data we have collected, they'll actually see what problems they have in their districts… So I think… now we'll be able to manage mental health better… (Provincial Information Manager, South Africa)*.

However, our analysis of data from document reviews and interview transcripts revealed that the use of mental health information was still limited by district level management committees in South Africa, compared with the use of information from other health programmes. Nevertheless, the respondents reflected that data gathered in the district was informally used by mental health coordinators and fed back to the mental health coordinators at provincial levels.

The MHIS strengthening that was perceived, led to some unintended negative effects. For example, the respondents reflected that the workload of clinical staff, particularly consulting room nurses, increased in each of the three hospitals in Ghana. Although not unexpected, this finding also raises a possible need for further refining the data collection forms and processes, to keep increases in workload to a minimum (or perhaps even to reduce the workload by making the data collection and processing more efficient). According to the respondents, the benefits of the improved systems, however, outweighed the negative effects.

### Key influences on the MHIS intervention

Different factors were reported to influence the design and implementation stages of MHIS strengthening in Ghana and South Africa. These are summarized in Table 3 and represent three broad categories identified by researchers in the analysis: resources issues (such as staff time and skills), working approaches/principles (which are the processes involved in integrating mental health into existing systems such as degree of consultation and communication) and wider contextual factors such as degree of political will, socio-economic factors and cultural constraints towards mental health leading to its low priority.

**Table 3.** Key influences on the mental health information systems (MHIS) design and implementation in the two countries

MHIS stage	Key positive (+) and negative (−) influences	
	South Africa	Ghana
Design	(1) Adequate time and expertise of the researchers (+) (2) User-friendliness (+) (3) Adequate consultations with stakeholders and increased ownership (+) (4) Ownership (+) (5) Integration of MHIS within district health information system (+) (6) Low priority of mental health (−)	(1) Expertise of researchers and support system (+) (2) Endorsement through: (+) (3) Ownership of the project (+) (4) Adequate consultations (+) (5) Low priority of mental health (−)
Implementation	(1) Adequate staff motivation (±) (2) Resources and support system (±) (3) Varied communication among staff (−) (4) Lack of culture of information use (−) (5) Low priority of mental health (−)	(1) Resources (+/−) Procurement of new computers, (+) Availability of forms, poor internet accessibility and data security issues (**−**) (2) Motivation of staff (±) (3) Low priority of mental health (**−**) (4) Poor communication (**−**) (5) Low confidence in research projects (**−**)

**Fig. 1.**
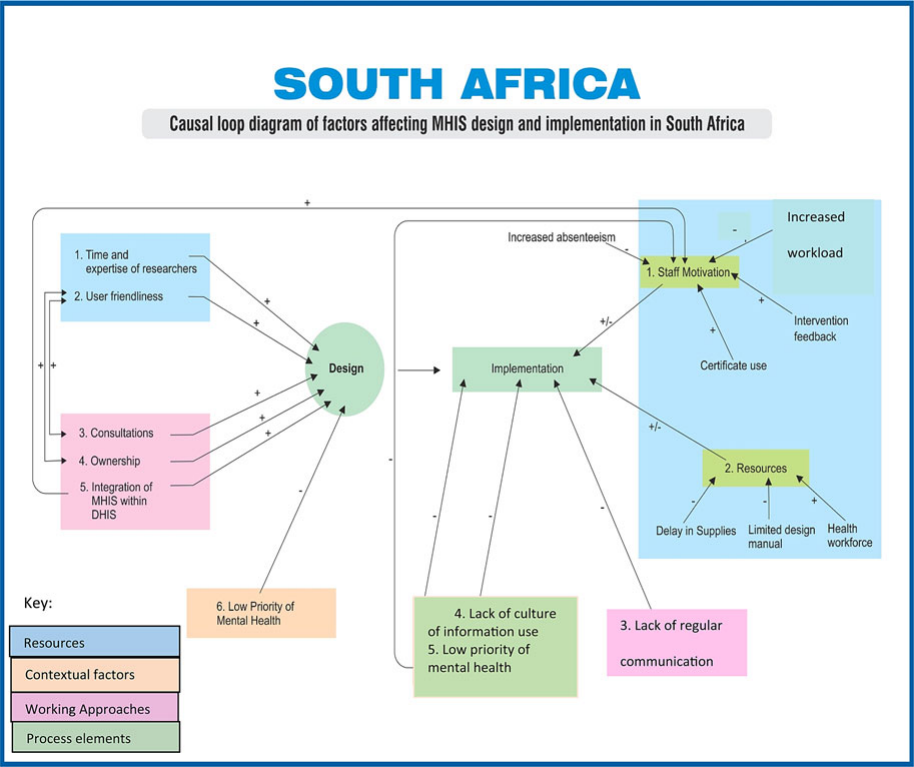
Causal loop diagram: key influences on mental health information systems (MHIS) design and implementation in South Africa.

**Fig. 2.**
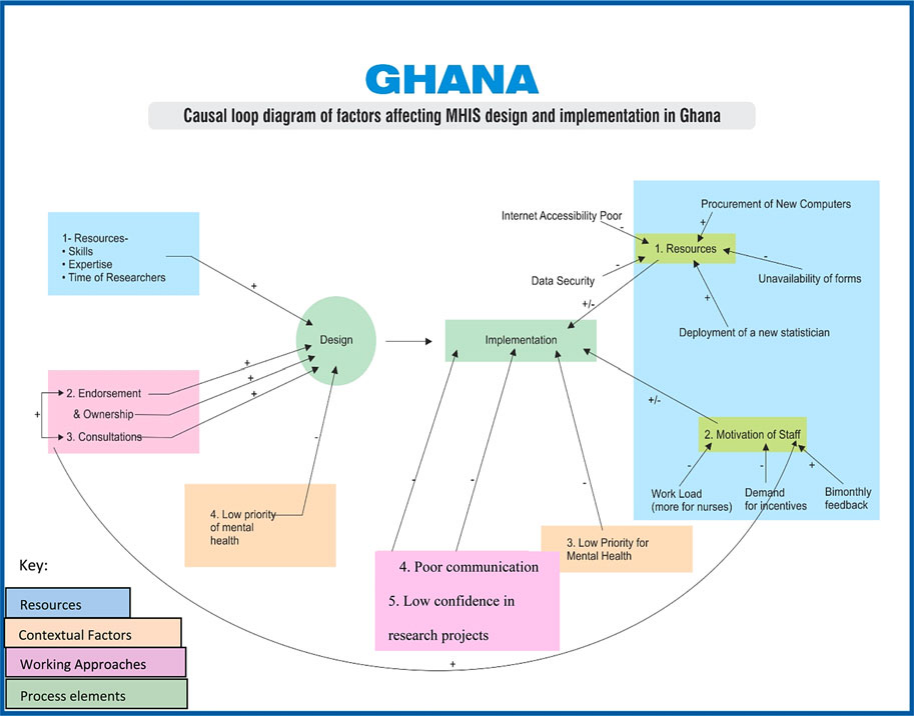
Causal loop diagram: key influences on mental health information systems (MHIS) design and implementation in Ghana.

Our analysis of documents and interview data identified different positive and negative influences of these factors on the MHIS design and implementation. These are illustrated in Figs 1 and 2 for South Africa and Ghana respectively, using a simplified version of causal loop diagrams method.

### Key influences in MHIS design

As illustrated in Figs 1 and 2, the respondents’ perceptions of positive influences in both countries included availability of different resources and continuous consultations. In South Africa, the respondents also referred to the integration of MHIS within DHIS as a positive influence. In both countries the low priority of mental health as a policy issue was perceived to be a constraint. These influences are set out next in more detail.

#### Resources

All respondents reflected that adequate staff expertise and sufficient time spent by different experts in the field helped to ensure the appropriate design of the new system. In South Africa, the main resource-related issue which positively affected the MHIS design, identified in both documents and interview data, was the establishment of task teams involving 5–6 members with different expertise, comprising researchers and information management staff, mental health programme management and monitoring and evaluation staff.

#### Consultations

Continuous consultations with, and involvement of, key stakeholders during the MHIS design in Ghana and South Africa were perceived to be crucial for ensuring their ownership and commitment to the project, as one respondent reflected:

*I think in terms of what worked well is the involvement of …all the important role-players from the doctors and the programme managers and the coordinators at the district level, as well as the doctors at the tertiary, at the hospital level. (Provincial Information Manager, South Africa)*.

In Ghana, analysis of interviews and documents revealed that consultations with key stakeholders were conducted throughout the design stage, including planning the changes to the existing system, incorporating ICD-10 and enhancing the capacity to implement MHIS. It was evident in the data that consultations hence became a continuing factor enabling communication throughout intervention.

#### Low priority of mental health

One contextual constraint to MHIS design, referred to by the respondents in both countries, was the low priority of mental health as a policy issue when compared, for example, with other diseases such as communicable diseases. The low priority of mental health in both Ghana and South Africa was also evident in the documents reviewed from both countries. According to the respondents, this low priority of mental health also contributed to limited interactions between information management staff and clinical staff, who according to a district information officer in South Africa were:

*“…based in same offices but… are almost a world apart…”*.

The stakeholders reported opinions that this relatively low priority of mental health contributed to a challenge of integrating mental health indicators into routine HMIS in Ghana. On reflection, this is perhaps less of an information technology-related challenge and relates more to allocation of resources on the basis of perceived policy priorities. In other words, the general health planners and information managers involved in wider HMIS may not have seen the value in spending their time and resources for adding the MHIS indicators into the overall dataset.

#### Key influences in MHIS implementation

The MHIS implementation was also affected by different influences. As shown in Figs 1 and 2, while clear constraints were easily identifiable, similar factors were reported as both facilitating and constraining the implementation. In Ghana, the key stakeholders reported their low confidence in research projects, possibly reflecting the lack of clear benefits from similar initiatives in the past. In South Africa, the lack of general culture of information used in management decisions was reported as a constraint, though further probing revealed that this phenomenon is not specific to mental health.

The views about positive and negative influences were common in the two countries, and included availability of resources including staff motivations, degree of communication and low priority of mental health. These influences are set out next.

#### Resources

Unlike the design stage where the respondents identified mostly positive influences, the implementation stage was associated by the respondents with different positive and negative influences in Ghana and South Africa. As one would normally expect, availability of resources is likely to catalyze the implementation, whereas their absence is likely to be a constraint. In Ghana, procurement of new computers, recruitment of records staff and deployment of new statisticians in some hospitals helped to implement the new system. However, inconsistent internet connectivity, inadequate storage files and data security concerns (e.g. outdated antivirus software), were examples of specific implementation challenges the stakeholders faced in Ghana. According to the respondents, the shortage of paper for printing the data collection forms, due to delays in supplies from provincial level in South Africa, led to delays in the data collection. However, the data collection still continued where it was feasible, largely due to motivated staff.

The influence of the health workforce on MHIS implementation was found to be significant in both countries. As reflected by one respondent consideration of importance of health workforce represents a somewhat neglected area, compared with the other components of the system.

*I think we just sort overlooked the area that you know for those indicators to be …for us to get that data into the system, there must be people who must really put in the data…so I think we sort of neglected that component. (Provincial Information Manager, South Africa)*.

In South Africa, different respondents reflected on the motivating nature of incentives for improving data collection, such as certificates to facilities, which performed well during the training:

*I think that giving out the certificates was a very big motivation… I think it had a great impact… (District Mental Health Coordinator, South Africa)*.

In Ghana, continued monitoring of implementation by the research team and other stakeholders helped establishing the system of bi-monthly feedback to the hospital staff containing information on patient data. According to the respondents and the documents, this feedback contributed to increase in staff motivation and led to better appreciation of value of good information for management and planning decisions, as one respondent reflected in Ghana:

*Now the data we collect is more accurate, because the disorder classification is more and they can be easily fit into the classifications. It is not like in the past, where we had to force the other disorders into the classification of other mental disorders”-Recorder, Psychiatric Hospital, Monitoring visit Ghana*.

However, the clinical health staff in both South African provinces sometimes felt the data collection under the new MHIS considerably increased their workload.

*We are still having problems with some of the sisters …they actually say they don't have time to actually fully implement the registers but that their statistics could be fairly accurate. (District Mental Health Coordinator, South Africa)*.

Similarly, different respondents in Ghana referred to increased staff workload as the most challenging issue during the implementation of the new MHIS: for example, dual filling of the forms on paper and computers led to 3 months of data entry back log and the instances of incomplete data collection during the initial stages of MHIS implementation.

In Ghana, the perceived increase in staff workload was in contrast with their expectations that the intervention would mostly use time of the researchers, and not of their own time. This appears to have led to some resistance, and less trust in research projects, reflected in the documents such as meeting minutes, but may also reflect a lack of established culture of information use in decision making, which was identified by the respondents as a constraint for MHIS implementation.

#### Communications

Unlike the design stage, the lack of regular communication was identified by the stakeholders as a constraint to MHIS implementation in both countries. For example, some respondents reflected that hospital managers, clinicians and information managers could have been involved more as the intervention progressed in South Africa. In Ghana, each hospital, perhaps understandably, adopted their own flows of information. However, there was lack of regular communication between different departments, for example, resulting in complaints by pharmacy departments and wards for not receiving the information forms in time.

#### Low priority of mental health

Similar to the design stage, the need for improved prioritizing of mental health as a policy issue was also felt during the implementation of the intervention in both countries. For example, in South Africa the respondents reflected that mental health was being neglected by senior management staff, leading to limited availability of resources and potentially contributing to low staff motivation:

*…. but I think in terms of allocating budget, there is less money that is allocated for mental health, which is a crucial thing that needs to be addressed from national to local level (District Manager, South Africa)*.

## Discussion

Reflecting on the key influences identified by stakeholders across the two stages together, more influences were identified on MHIS implementation, compared with the design stage. However, the factors identified during both stages represented similar three categories (resource issues, working approaches and wider context). Furthermore, analysis shows that the MHIS design stage had more positive influences as compared with the implementation stage. Key positive influences included consultations and working principles, whereas the key negative influences included poor staff motivation at facility level for data collection, lack of culture of information use in decisions and less synergy between MHIS and other staff tasks.

Our findings illustrate different influences on the MHIS design and implementation which are similar with other studies. For example, a study in Malawi identified the importance of staff motivation for strengthening HMIS (Chaulagai *et al*. 2005). Similarly in Uganda, the organizational issues, involving restructuring the role of medical records officer at district and facility levels, were found to be important (Gladwin *et al*. 2003). Consultations can increase participation and ownership by these stakeholders (WHO, 2005): in the USA close collaboration between programme evaluators, policy makers and clinical leadership enabled enhancing the role of technology in the implementation of mental health services policy requirements in the Veterans Health Administration Trafton *et al*. (2013).

In South Africa, the strengthening of MHIS was integrated within wider DHIS in two study provinces. This approach is likely to be a more sustainable solution as opposed to stand-alone and parallel systems addressing the needs of individual projects or vertical programmes (HMN, 2008). The challenge of this approach, however, is around adding mental health indicators to an already long and established list of other health indicators, and resistance from primary care staff to taking on new mental health responsibilities. On the other hand, in Ghana focusing on the three psychiatric hospitals allowed more targeted support. There was clearly less of a need to convince clinicians and information officers within three psychiatric hospitals of the importance of mental health. The challenges of focusing on specialist setting include technical difficulties in establishing consistent indicators for aggregating data, and bringing about changes in the behaviour of clinicians and records staff. On reflection, there are advantages to each approach and perhaps a combination of both approaches would be appropriate for many contexts to ensure the balance between sustainability due to integration and targeted support due to focus on a specific health area such as the mental health.

The importance of human resources is often underestimated in designing and implementing HIS (WHO, 2004). Staff motivation is particularly important in retaining staff and improving their performance (Henderson & Tulloch, 2008). Unless motivated, the increased turnover can lead to poor quality of the data collected (WHO, 2004). Conversely, well-motivated staffs are more likely to improve data quality.

The wider context, similar to other studies, is important in ensuring the success of such interventions. Two particular contextual issues are worth emphasizing. First, is a relatively low importance of mental health within existing national health priorities in these countries (Bird *et al*. 2011). Second, is the importance of appropriate capacity to plan and implement complex interventions while ensuring ownership of, and continuous support to, the project (Lafond *et al*. 2002). In our case the partnership of researchers and key stakeholders served as the backbone for the MHaPP project including the MHIS interventions (Mirzoev *et al*. 2012). However, less confidence in research projects of Ghanaian hospital staff and the preferences for regular communication with researchers during implementation raise a question of sustainability of the MHIS interventions following the withdrawal of research teams.

More influences were identified during implementation than design. This may be a reflection of a wider array of influences in implementing complex health systems interventions. MHIS design had more positive influences but negative influences – such as lack of design manuals and poor culture of information in South Africa, lack of motivation of the staff and poor flow of registration forms through the Ghanaian hospitals – were also identified, suggesting the need to consider these issues in exploring feasibility of similar interventions.

Our analysis reveals that similar factors can have both negative and positive influences so the distinction between positive and negative influences may not always be clear cut. Examples of such factors include staff motivations in Ghana and resources and support system in South Africa during the MHIS implementation. These factors can be perceived on a continuum where, at one end, the existence of motivated staff can positively catalyze the process of MHIS implementation, whereas at the other end, the absence of motivated personnel can constrain quality and comprehensiveness of data collection.

The MHIS design and implementation, although separated in this paper, are related parts of a single process. Often, implementation challenges and successes are due to their consideration or otherwise in the design stage. This may explain the similarity of some influencing factors such as low priority of mental health, which cuts across both MHIS design and implementation.

Different influences appear to be related within, and across, the MHIS design and implementation and may reinforce or negate each other thus leading to the multiplier or minimization effects as shown in the casual loop diagrams. For example, in Ghana the degree of staff consultations in the intervention design is likely to affect the degree of their ownership of the intervention and, ultimately, their motivation to accurately collect data. Similarly in South Africa low priority of mental health affects the motivation of staff negatively, whereas integration of MHIS in DHIS is likely to have positive implications on staff motivation. In both Ghana and South Africa, clinical staff at facility level felt less motivated and less dedicated towards MHIS tasks than towards their clinical work – an important aspect that requires consideration during designing of MHIS. Also, consultations with key relevant stakeholders emerged from our analysis as a crucial factor determining the success of the project through positive implications on staff motivation and ownership of the intervention.

Three implications can be derived from our study for the future MHIS strengthening interventions in these two countries, and other similar contexts. First, adequate consultations with key stakeholders during MHIS design and ensuring staff motivation to accurately collect data are likely to be particularly important in ensuring the success of similar interventions. Second, design and implementation stages are related and better consideration of implementation challenges during the intervention design should improve the feasibility of MHIS strengthening and contribute to the sustainability of achieved changes. Last, different influencing factors are interrelated, can lead to potential multiplier effects and are likely to be affected by the relative priority of the mental health programmes within wider health systems; therefore, recognition of this complexity is important in planning and implementation of similar initiatives.

### Study limitations

We acknowledge several limitations to our study. First, in this paper we report mostly the perceptions of key stakeholders and not the results of direct assessment of effectiveness of MHIS strengthening. While this can be regarded as a limitation, our approach is driven by our focus on the identification of key influences which is complementary to future research and is identified by exploring the perceptions of different stakeholder groups, given the methodological challenges associated with attributing specific influences on the intervention design and implementation.

Further research can be appropriate to compare stakeholder perceptions with direct evaluation of effectiveness of MHIS strengthening. Second, research participants were also participants in the intervention in some instances, and this may have led to bias in their responses. While it was impossible to completely avoid such bias, triangulation between the views of the different stakeholder groups and between analysis of interviews and documents allowed us to minimize this. Third, the researchers in the countries were also involved in designing and implementing the interventions, and this may have biased their evaluation. The triangulation between the different methods and information sources referred to earlier, and analysis of data by more than one researcher, helped us to minimize researcher bias and ensure validity of our results. Further research is needed to evaluate the design and implementation of information systems for mental health, particularly in African countries.

## Conclusion

In this paper we reported the main effects of, and key influences on, MHIS strengthening as perceived by the key stakeholders in the two African countries. Influences on the design and implementation of MHIS interventions in Ghana and South Africa relate to resources, working approaches (including degree of consultations during the design stage and communication during implementation stage) and the low priority of mental health. Three implications are proposed to inform future MHIS strengthening interventions in these and other similar countries: enhancing consultations during the design stage, improved consideration of implementation challenges during the design stage and better recognition of multiple and complex relations between different influences in the planning and implementation of similar initiatives.

## References

[ref1] AbouZahr C, Boerma T (2005). Health information systems: the foundations of public health. Bulletin of the World Health Organization 83, 578–583. 16184276 PMC2626318

[ref2] Bird P, Omar M, Doku V, Lund C, Nsereko JR, Mwanza J (2011). Increasing the priority of mental health in Africa: findings from qualitative research in Ghana, South Africa, Uganda and Zambia. Health Policy and Planning 26, 357–365. 21147845 10.1093/heapol/czq078

[ref3] Bloom D, Cafiero ET, Jané-Llopis E, Abrahams-Gessel S, Bloom LR, Fathima S, Feigl AB, Gaziano T, Mowafi M, Pandya A, Prettner K, Rosenberg L, Seligman B, Stein AZ, Weinstein C (2012). “The Global Economic Burden of Non communicable Diseases,” PGDA Working Papers 8712, Program on the Global Demography of Aging.

[ref4] Brender J, de Keizer N, Nykänen P, Rigby M, Ammenwerth E (2013). STARE-HI – statement on reporting of evaluation studies in health informatics. Explanation and elaboration. Applied Clinical Informatics 4, 331–358. 24155788 10.4338/ACI-2013-04-RA-0024 PMC3799207

[ref5] Chaulagai CN, Moyo C, Koot J, Moyo H, Sambakunsi T, Khunga F, Naphini P (2005). Design and implementation of a health management information system in Malawi: issues, innovations and results. Health Policy and Planning 20, 375–384. 16143590 10.1093/heapol/czi044

[ref6] Doku V, Ofori-Atta A, Akpalu B, Read U, Osei A, Ae-Ngibise K, Awenva D, Lund C, Flisher AJ, Petersen I, Bhana A, Bird P, Drew N, Faydi E, Funk M, Green A, Omar M (2008). The Mental Health and Poverty Project: Phase 1. Country Report: A Situation Analysis of Mental Health Policy Development and Implementation in Ghana. p. 345.

[ref7] Flisher AJ, Lund C, Funk M, Banda M, Bhana A, Doku V, Drew N, Kigozi FN, Knapp M, Omar M, Petersen I, Green A (2007). Mental health policy development and implementation in four African countries. Journal of Health Psychology 12, 505–516. 17440000 10.1177/1359105307076237

[ref9] Garrib A, Stoops N, McKenzie A, Dlamini L, Govender T, Rohde J (2008). An evaluation of the district health information system in rural South Africa. South African Medical Journal 98, 549–552. 18785397

[ref10] Gladwin J, Dixon RA, Wilson TD (2003). Implementing a new health management information system in Uganda. Health Policy and Planning 18, 214–224. 12740326 10.1093/heapol/czg026

[ref11] Health Metrics Network (2008). Framework and Standards for Country Health Information Systems. 2nd edn. Health Metrics Network, World Health Organization: Geneva.

[ref12] Henderson L, Tulloch J (2008). Incentives for retaining and motivating health workers in Pacific and Asian countries. Human Resources for Health 6, 18. 18793436 10.1186/1478-4491-6-18 PMC2569066

[ref13] Krishnan A, Nongkynrih B, Yadav K, Singh S, Gupta V (2010). Evaluation of computerized health management information system for primary health care in rural India. BMC Health Services Research 10, 310. 21078203 10.1186/1472-6963-10-310 PMC2996385

[ref14] LaFond A, Brown L, Macintyre K (2002). Mapping capacity in the health sector: a conceptual framework. International Journal of Health Planning and Management 17, 3–22. 11963442 10.1002/hpm.649

[ref15] Lund C, Kleintjes S, Campbell-Hall V, Mjadu S, Petersen I, Bhana A, Kakuma R, Mlanjeni B, Bird P, Drew N, Faydi E, Funk M, Green A, Omar M, Flisher AJ (2008). Mental Health Policy Development and Implementation in South Africa: a Situation Analysis. Phase 1 Country Report. Mental Health and Poverty Project: Cape Town, South Africa.

[ref16] Lund C, Kleintjes S, Kakuma R, Flisher AJ (2009) Public sector mental health systems in South Africa: inter-provincial comparisons and policy implications. Social Psychiatry and Psychiatric Epidemiology 45, 393–404. 19506789 10.1007/s00127-009-0078-5

[ref17] Lund C, Flisher AJ (2003). Community/hospital indicators in South African public sector mental health services. Journal of Mental Health Policy and Economics 6, 181–187. 14713725

[ref19] MHaPP (2010). Mental Health Information System Pilot Project Report: KwaZulu-Natal. Mental Health and Poverty Project. University of Cape Town: Cape Town, South Africa.

[ref18] MHaPP (2010 *a*). Mental health policy development and implementation in four African countries. Final Report. MHaPP Research Programme Consortium: Cape Town.

[ref20] Miles M, Huberman M (1994). Qualitative Data Analysis: A Sourcebook of New Methods, 2nd edn Sage Publications: Beverly Hills, CA.

[ref21] Mirzoev T, Omar M, Green A, Bird P, Lund C, Ofori-Atta A, Doku V (2012). Research-policy partnerships – experiences of the mental health and poverty project in Ghana, South Africa, Uganda and Zambia. Health Research Policy and Systems 10, 30. 22978604 10.1186/1478-4505-10-30 PMC3542094

[ref22] Murray CJ (2012). Disability-adjusted life years (DALYs) for 291 diseases and injuries in 21 regions, 1990–2010: a systematic analysis for the global burden of disease study 2010. Lancet 380, 2197–2223. 23245608 10.1016/S0140-6736(12)61689-4

[ref23] Nykänen P, Brender J, Talmonc J, Keizer N, Rigbye M, Beuscart-Zephir MC, Ammenwerth E (2011). Guideline for good evaluation practice in health informatics (GEP-HI). International Journal of Medical Informatics 80, 815–827. 21920809 10.1016/j.ijmedinf.2011.08.004

[ref24] Odhiambo-Otieno GW (2005). Evaluation criteria for district health management information systems: lessons from the Ministry of Health, Kenya. International Journal of Medical Informatics 74, 31–38. 15626634 10.1016/j.ijmedinf.2004.09.003

[ref26] Ofori-Atta A, Mirzoev T, Mensah-Kufuor A, Osei A, Dzadey A, Armah-Aloo K, Atweam KD (2012). Experience of strengthening the mental health information system in Ghana's three psychiatric hospitals. reader in psychiatry In Changing Trends in Mental Health Care and Research in Ghana (ed. A. Ofori-Atta and S. Ohene), p. 22. University of Ghana 60th Anniversary Series: Accra, Ghana, 22.

[ref27] Ofori-Atta A, Read UM, Lund C (2010). A situation analysis of mental health services and legislation in Ghana: challenges for transformation. African Journal of Psychiatry 13, 99–108. 20473470 10.4314/ajpsy.v13i2.54353

[ref28] Omar MA, Green AT, Bird PK, Mirzoev T, Flisher AJ, Kigozi F, Lund C, Mwanza J, Ofori-Atta AL (2010). Mental health policy process: a comparative study of Ghana, South Africa, Uganda and Zambia. International Journal of Mental Health Systems 4, 24. 20678205 10.1186/1752-4458-4-24 PMC2923107

[ref29] Ritchie J, Spencer L (1994). Qualitative data analysis for applied policy research In Analyzing Qualitative Data (ed. A. Bryman and R. Burgess), p. 9. Routledge: New York, 9.

[ref31] Smith M, Shirin M, Adebusoye A, Mwele LM, Edwin M (2008). Integrating health information system in Tanzania: experience and challenges. Electronic Journal of Information System in Developing Countries 33, 1–21.

[ref32] Trafton JA, Greenberg G, Harris AH, Tavakoli S, Kearney L, McCarthy J, Blow F, Hoff R, Schohn M (2013). VHA mental health information system: applying health information technology to monitor and facilitate implementation of VHA Uniform Mental Health Services Handbook requirements. Medical Care 51(3 Suppl. 1), S29–S36. 23407008 10.1097/MLR.0b013e31827da836

[ref33] WHO (2004). Developing Health Management Information Systems. A Practical Guide For Developing Countries. World Health Organization. Regional Office for the Western Pacific: Manila, Philippines.

[ref34] WHO (2005). Mental Health Policy and Service Guidance Package. Mental Health Information Systems. World Health Organisation: Geneva.

[ref36] WHO (2010). Mental Health and Development: Targeting People with Mental Health Conditions as a Vulnerable Group. World Health Organization: Geneva.

